# Are past and future symmetric in mental time line?

**DOI:** 10.3389/fpsyg.2015.00208

**Published:** 2015-02-26

**Authors:** Xianfeng Ding, Ning Feng, Xiaorong Cheng, Huashan Liu, Zhao Fan

**Affiliations:** ^1^Key Laboratory of Adolescent Cyberpsychology and Behavior, Ministry of EducationWuhan, China; ^2^School of Psychology, Central China Normal UniversityWuhan, China

**Keywords:** mental time line, asymmetry, STARC effect, distance effect

## Abstract

A growing body of evidence has suggested that time, from early to late, or from past to future, was represented in a spatially oriented mental time line. However, little is known about its characteristics. The present study provided the first empirical evidence to explore the symmetry of spatial representations of past and future in the mental time line. Specifically, we compared the Spatial-Temporal Association Response Codes (STARC) effects and distance effects of past and future in four experiments. Results showed that for near past and near future, STARC effects were similar (Experiment 1). For distant past, the STARC effect was significant, but not for distant future (Experiment 2). Furthermore, the distance effect in the past was significantly stronger than in the future (Experiments 3, 4). These findings supported the idea that time points are not evenly distributed in mental time line. Spatial representations of the past and the future are asymmetric, and the spatial representation of past seems stronger than future. The logarithmic pattern of internal spatial representation of past or future is also discussed.

## Introduction

Human beings often represent abstract concepts in concrete visual-spatial images. The spatial representation of number is a typical instance. It was suggested that numbers are represented in a continuous mental number line based on the extensive research on the Spatial-Numerical Association of Response Codes (SNARC) effect (Dehaene et al., 1993; Fischer et al., 2003; Schwarz and Keus, 2004; Hubbard et al., 2005; Nuerk et al., 2005a,b). Small numbers are represented at the left side of the line, while large numbers are represented at the right side. Time is also tightly connected with space. Specifically, researchers recently observed a SNARC like effect with time, which was labeled as the Spatial-Temporal Association of Response Codes (STARC) effect (Ishihara et al., 2008; Vallesi et al., 2008). Therefore, time was analogically thought to be represented in a mental time line similar to the mental number line. In other words, time is represented in a continuous spatial line with a left-to-right orientation, where time flows from early to late, or from past to future (Bonato et al., 2012).

The mental time line hypothesis was supported by three categories of spatial-temporal congruency effects. The first type of congruency effect was based on temporal duration or interval. Vallesi et al. (2008) found that left responses were faster when associated with a short duration, while right responses were faster when associated with a long duration. The authors thought this compatible effect was a result of the spatial representation of elapsing time. When a temporal duration has to be estimated, elapsing time may be represented progressively from the left to the right. Then a short duration would be represented relatively to the left, and a long duration relatively to the right (See also Vallesi et al., 2011; Fabbri et al., 2012). Furthermore, duration estimation or judgment can also be influenced by spatial attention. Time duration would be underestimated when attention was directed to the left space, and be overestimated when attention was directed to the right space (Vicario et al., 2007; Frassinetti et al., 2009). A short duration was responded faster when a visual prime was in the left space, whereas a long duration was responded faster when a visual prime was in the right space (Di Bono et al., 2012). These findings suggested that elapsing time was represented in a mental time line from the left to the right.

The second type of congruency effect was based on temporal order. Santiago et al. (2010) found a space-time congruency effect when meaningful event sequences were presented by means of naturalistic movie clips or picture sequences. Order judgments between two events were faster when the left hand was used to respond “before” and the right hand to respond “after” than when responded with the opposite mapping (see also Fuhrman and Boroditsky, 2010; Boroditsky et al., 2011). However, inherent and logical associations between successive stimuli may be confounded with temporal order in these studies. Some researchers found the STARC effect when using stimuli without logical or internal links. Previtali et al. (2010) used nine words to explore the congruency effect of order and space in a serial learning paradigm. After an over-learned training phase, these nine words showed a SNARC similar effect for both order-relevant and order-irrelevant tasks. Moreover, a STARC effect based on mere temporal order was also found in working memory paradigms (Ding et al., 2015). These findings indicated that we could represent temporal order information in a spatial line.

The third type of congruency effect was based on abstract time words. Gevers et al. (2003, 2004) found that early months of a year or early days of a week were responded faster with the left key, whereas late months or days were responded faster with the right key. In addition, words referring to the past were responded faster with the left hand; words referring to the future were responded faster with the right hand (Santiago et al., 2007). This effect was found in both visual and auditory modalities (Lakens et al., 2011; Kong and You, 2012). Furthermore, time words can shift attention. Words related to the past can shift attention to the left and words related to the future can shift attention to the right in priming tasks (Weger and Pratt, 2008, 2009; Ouellet et al., 2010a). These findings indicated that the abstract concept of time could also be represented in a spatially oriented mental time line. Past is at the left side of this line, and future is at the right side of this line. Time flows from past (left) to future (right) in the mental time line.

Taken together, three categories of evidence strongly supported that time can be represented in a mental time line. Time, from early to late, or from past to future, appears to be represented in a left-to-right spatially continuous line. Previous studies have provided a lot of evidence for the existence of the mental time line, however, little is known about its characteristics. Are time points distributed evenly in this mental time line? Specifically, are past and future symmetric in the mental time line? The past is time we have actually experienced while the future is time that we have never experienced. Could this difference in reality for past and future lead to different spatial representations? A temporal asymmetry of past and future was suggested by evidence in some other paradigms. Representations of past events were associated with more specific details than representations of future events (D'Argembeau and van der Linden, 2004, 2006; Addis and Schacter, 2008; Wang et al., 2011), and future events were more prototypical than past events (Kane et al., 2012). Thus, we hypothesized that the spatial representations of past and future with the same temporal distance from the present are not identical but asymmetric in the mental time line. Examining symmetry would provide the first empirical evidence of characteristics of the mental time line, which is important to the construction of a theory of the spatial representation of time. Moreover, it will enhance the understanding of difference or similarity of past and future from the aspect of spatial linear representations and further provide a more specific spatial frame for past-future related theories.

To investigate this issue, we compared the spatial representations of past and future in the mental time line. According to previous studies, the STARC effect is the most important index of the spatial representation of time. Thus, we explored the symmetry of past and future in the mental time line by comparing the STARC effects of past and future. Another typical index of the spatial representation is the distance effect, in which the distance discrimination of two points located on a spatial line would be faster when the two points are far from each other than when they are near from each other (Moyer and Landauer, 1967; Dehaene et al., 1990; Dehaene, 1997). So we also compared the distance effects between past and future. We hypothesized that if the past and future are symmetric in the mental time line, there should be no differences on STARC effects and distance effects between past and future.

## Experiment 1

Experiment 1 was designed to examine whether the STARC effects for near past (yesterday) and near future (tomorrow) were different. If the STARC effects were the same, it would support that spatial representations of near past and near future were symmetric in the mental time line.

### Methods

#### Participants

Thirty six undergraduate students (13 male and 23 female) from Central China Normal University participated in the experiment for course credits. All participants signed a consent form according to the requirements of Institutional Review Board of CCNU. They were 19.6 years old on average (range 18 to 21). All participants were naive to the purpose of the experiment.

#### Stimuli and apparatus

Sixteen Chinese time words were used, 8 referring to the past time of yesterday (e.g., yesterday morning, yesterday afternoon, yesterday evening, etc.), and the other 8 referring to the future time of tomorrow (e.g., tomorrow morning, tomorrow afternoon, tomorrow evening, etc.). Time for the two groups of words were the same except that past time was labeled with yesterday, and future time was labeled with tomorrow.

Participants viewed words on a 17-in. CRT screen (refresh rate 75 Hz and resolution 1280 × 1024 pixels) from a distance of 70 cm. The experiment procedure was programmed in Visual C++.

#### Experimental design

We used a 2 × 2 × 2 mixed design. A between-subjects factor was Type of Time Words (yesterday vs. tomorrow) and two within-subjects factors were Temporal Position (early vs. late), and Response Congruence (congruence vs. incongruence, congruence means early stimuli responded with the left key and late stimuli responded with the right key; incongruence means early stimuli responded with the right key and late stimuli responded with the left key). Response times (RTs) and accuracy rates were dependent variables.

#### Procedure

Half of participants took part in the past or yesterday condition and the other half in the future or tomorrow condition. In the past condition, a trial started with a central fixation cross, lasting for 500 ms. Following that cross, a time word of yesterday was presented for 300 ms. Participant were required to judge whether the time of word was earlier or later than yesterday noon. For example, yesterday evening was later than yesterday noon. In one session, the participant pressed the left key (left arrow on the keyboard) if earlier and pressed the right key (right arrow on the keyboard) if later. In the other session participants responded in the opposite way. The order of the two sessions was counterbalanced across participants. The participants were required to respond as fast and accurately as possible using two fingers of the right hand only. After responses to stimuli, a 1000 ms blank separated one trial from another. Each session included 10 trials of practice and 4 blocks of 160 trials in the formal experiment.

In the future condition, the procedure was the same as in the past condition, except that the stimuli were time words of tomorrow and participants were required to judge whether the time was earlier or later than tomorrow noon.

#### Data analysis

Trials were treated as errors and discarded from the RT analyses if a response was made during the first 100 ms after the stimuli onset (anticipated responses), if the RT was slower than 2000 ms or no response was detected (delayed and null responses), or if the judgment was incorrect. RT outliers of correct trials (out of 3 standard deviations) were also filtered on a per-participant basis and excluded from analyses. A 2 × 2 × 2 repeated measures MANOVA was performed both for accuracy rates and mean RTs of correct trials.

### Results and discussion

**Figure 1:**
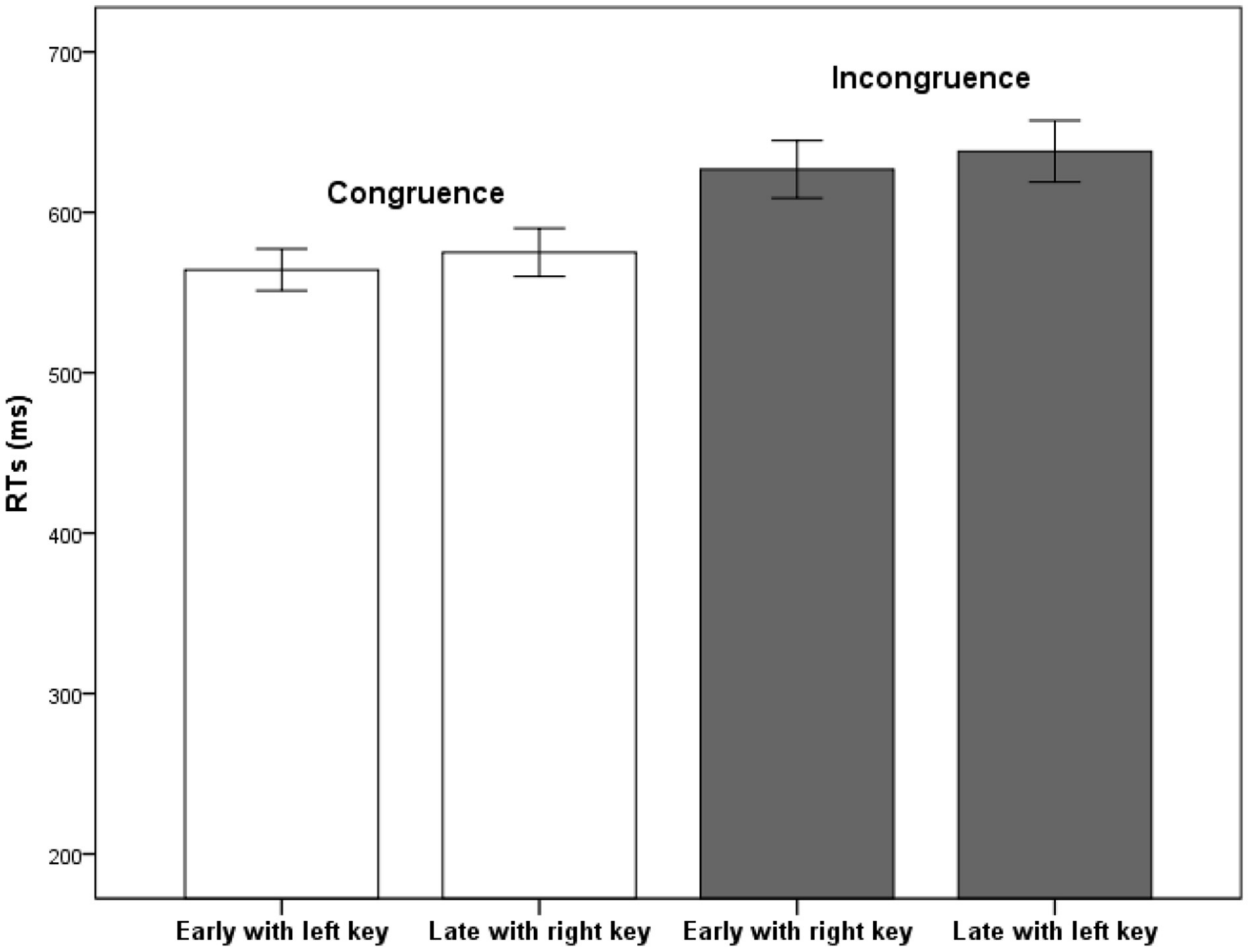
**Mean RTs in Experiment 1 as a function of response congruence and temporal position**. Error bars represent standard errors of the means.

The mean error rate in judging the time words was 2.31%. No significant effect was observed in the MANOVA concerning accuracy. The results of RTs indicated that the main effect of response congruence was significant (See Figure 1), *F*_(1, 34)_ = 29.33, *p* < 0.001, partial η^2^ = 0.46. The main effect of temporal position was significant, *F*_(1, 34)_ = 8.13, *p* < 0.01, partial η^2^ = 0.19. The main effect of type of time words was not significant, *F*_(1, 34)_ = 0.94, *p* > 0.05. The interaction between response congruence and type of time words was not significant, *F*_(1, 34)_ = 2.99, *p* > 0.05. The interaction between temporal position and response congruence was not significant, *F*_(1, 34)_ = 0.01, *p* > 0.05. The interaction between temporal position and type of time words was not significant, *F*_(1, 34)_ = 3.06, *p* > 0.05. The three-way interaction was not significant either, *F*_(1, 34)_ = 0.32, *p* > 0.05.

The results of Experiment 1 revealed a typical STARC effect. The RT of congruence (*M* = 569 ms, *SD* = 83) was significantly shorter than the RT of incongruence (*M* = 632 ms, *SD* = 110). In other words, early time of a day was responded faster with the left key and late time was responded faster with the right key. However, there was no significant interaction effect between response congruence and type of time words. The STARC effects were the same for near past (yesterday) and near future (tomorrow). This result is consistent with the idea that the spatial representations of past and future in near space are symmetric in the mental time line.

## Experiment 2

The STARC effects for near past and near future did not show any difference in Experiments 1, 2 was designed to further examine whether the STARC effects of distant past (last year) and distant future (next year) were different.

### Methods

#### Participants

Thirty six undergraduate students (16 male and 20 female) from Central China Normal University participated in the experiment for course credits. All participants signed a consent form according to the requirements of Institutional Review Board of CCNU. They were 20.1 years old on average (range 18 to 21). All participants were naive to the purpose of the experiment.

#### Stimuli and apparatus

Sixteen Chinese time words of festivals were used, 8 referring to past time of last year (e.g., Lantern Festival of last year, Labor Day of last year, National Day of last year, etc.) and the other 8 referring to future time of next year (e.g., Lantern Festival of next year, Labor Day of next year, National Day of next year, etc.). Times of the words were same except that past time was labeled with last year, and future time was labeled with next year.

#### Experimental design

The design was similar to the design in Experiment 1. The only change was the time words. Distant time words were used: last year vs. next year instead of yesterday vs. tomorrow.

#### Procedure

The procedure was the same as in Experiment 1. The task was to judge whether the time of word was earlier or later than July of last year or July of next year. For example, National Day of last year was later than last July. Half of participants took part in the past or last year condition and the other half in the future or next year condition.

#### Data analysis

Data analysis was the same as in Experiment 1.

### Results and discussion

**Figure 2:**
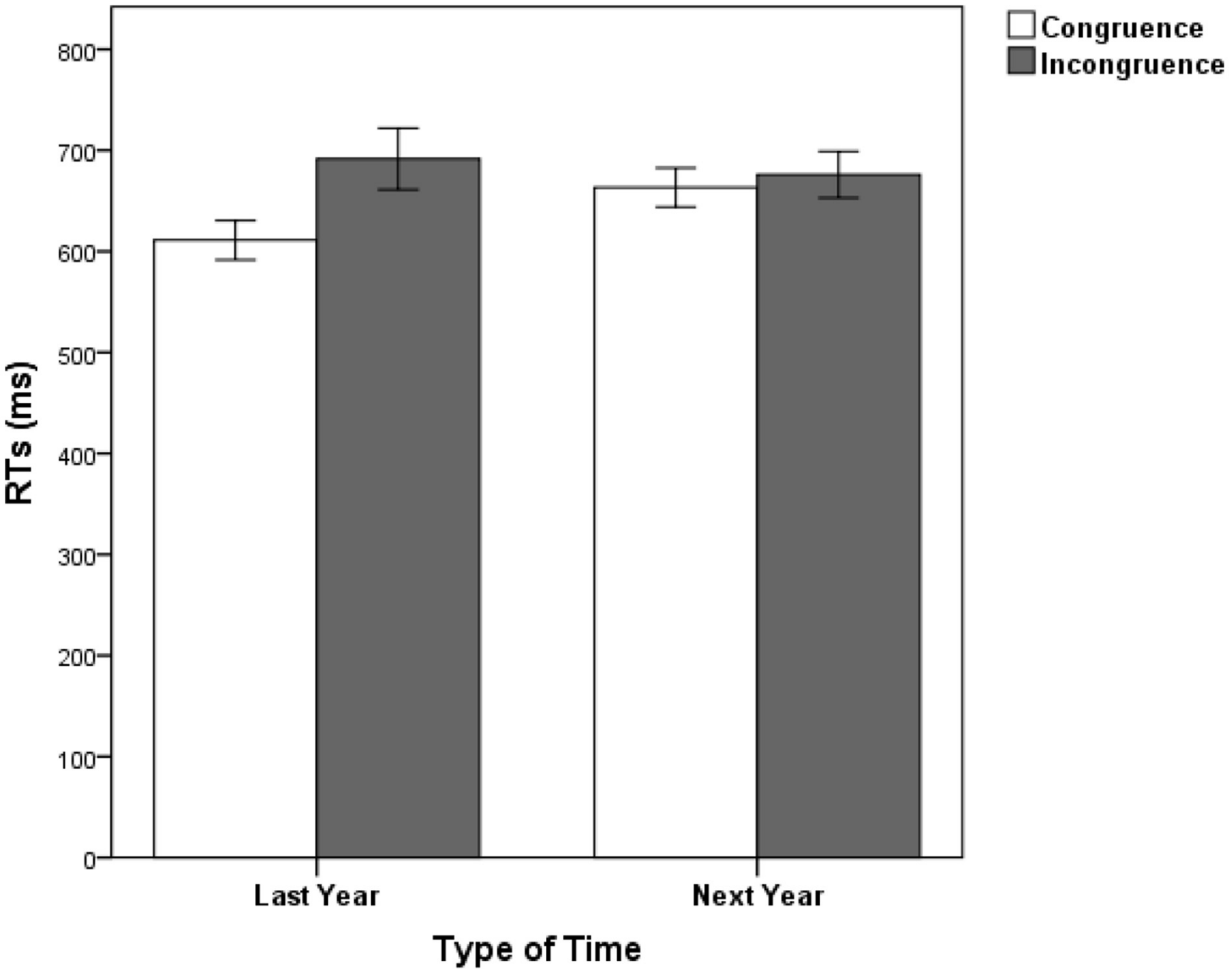
**Mean RTs in Experiment 2 as a function of type of time and response congruence**. Error bars represent standard errors of the means.

The mean error rate in judging the time words was 3.74%. No significant effect was observed in the MANOVA concerning accuracy. The results of RTs indicated that the only significant main effect was for response congruence, *F*_(1, 34)_ = 12.59, *p* < 0.001, partial η^2^ = 0.27. The interaction between response congruence and type of time words was significant, *F*_(1, 34)_ = 6.64, *p* = 0.014, partial η^2^ = 0.16 (See Figure 2). No other interaction was significant, *p*s > 0.05. Simple effect analysis revealed that the response congruence effect was significant only for last year, *F*_(1, 34)_ = 18.76, *p* < 0.001; but not significant for next year, *F*_(1, 34)_ = 0.47, *p* = 0.49.

The results of Experiment 2 revealed a significant STARC effect as in Experiment 1. However, there was an interaction between response congruence and type of time words. The STARC effect was only found in distant past (last year) condition, but not in distant future (next year) condition. Early times of last year were responded faster with the left key and late times of last year were responded faster with the right key, while this was not true for next year. Thus, it suggested that spatial representations of distant past and future were asymmetric in the mental time line, and the spatial representation of distant past was stronger than that of distant future.

## Experiment 3

The results of Experiments 1, 2 showed that spatial representations of past and future in the mental time line were symmetric in near space, but asymmetric in distant space. If this was the case, a specific distance of past or future (two time points from either near space or distant space with same temporal distance) might be represented asymmetrically in the mental time line. Consequently, the distance effect for past and future might be different. Experiment 3 was designed to further examine whether the distance effects of past and future were the same.

### Methods

#### Participants

Thirty six undergraduate students (14 male and 22 female) from Central China Normal University participated in the experiment for course credits. All participants signed a consent form according to the requirements of Institutional Review Board of CCNU. They were 21.2 years old on average (range 18 to 22). All participants were naive to the purpose of the experiment.

#### Stimuli and apparatus

Sixteen Chinese time words were used. Eight words were near distance time words, 4 of them referring to past time of yesterday (e.g., yesterday morning, yesterday evening, etc.) and the other 4 referring to future time of tomorrow (e.g., tomorrow morning, tomorrow evening, etc.). Eight words were far distance time words, 4 of them referring to past time of last year (e.g., Labor Day of last year, National Day of last year, etc.) and the other 4 referring to future time of next year (e.g., Labor Day of next year, National Day of next year, etc.).

#### Experimental design

We used a 2 × 2 × 2 mixed design. Three independent variables were temporal distance (near vs. far), between-subjects factor; type of time words (past vs. future), within-subjects factor; response congruence (congruence vs. incongruence, congruence means past time with left key and future time with right key; incongruence means past time with right key and future with left key), within-subjects factor. RTs and accuracy rates were dependent variables.

#### Procedure

Half of participants were in the far distance condition (last year and next year). A trial started with the central fixation cross, lasting for 500 ms. Following that cross, the time word of last year or next year was presented, lasting for 300 ms. Participants were required to judge whether the time of word was earlier or later than present. For example, National Day of last year was earlier than present. In one session, the participant pressed the left key (left arrow in keyboard) if earlier and pressed the right key (right arrow in keyboard) if later. In the other session participants were required to respond in the opposite way. The order of the two sessions was counterbalanced across participants. The participants were required to respond as fast and accurately as possible using two fingers of the right hand only. After responding to stimuli, a 1000 ms blank separated one trial from another. Each session included 10 trials of practice and 4 blocks of 160 trials in the formal experiment. The other half participants were in the near distance condition (yesterday and tomorrow). The procedure was the same as in the far distance condition.

#### Data analysis

Data analysis was the same as in Experiment 1.

### Results and discussion

**Figure 3:**
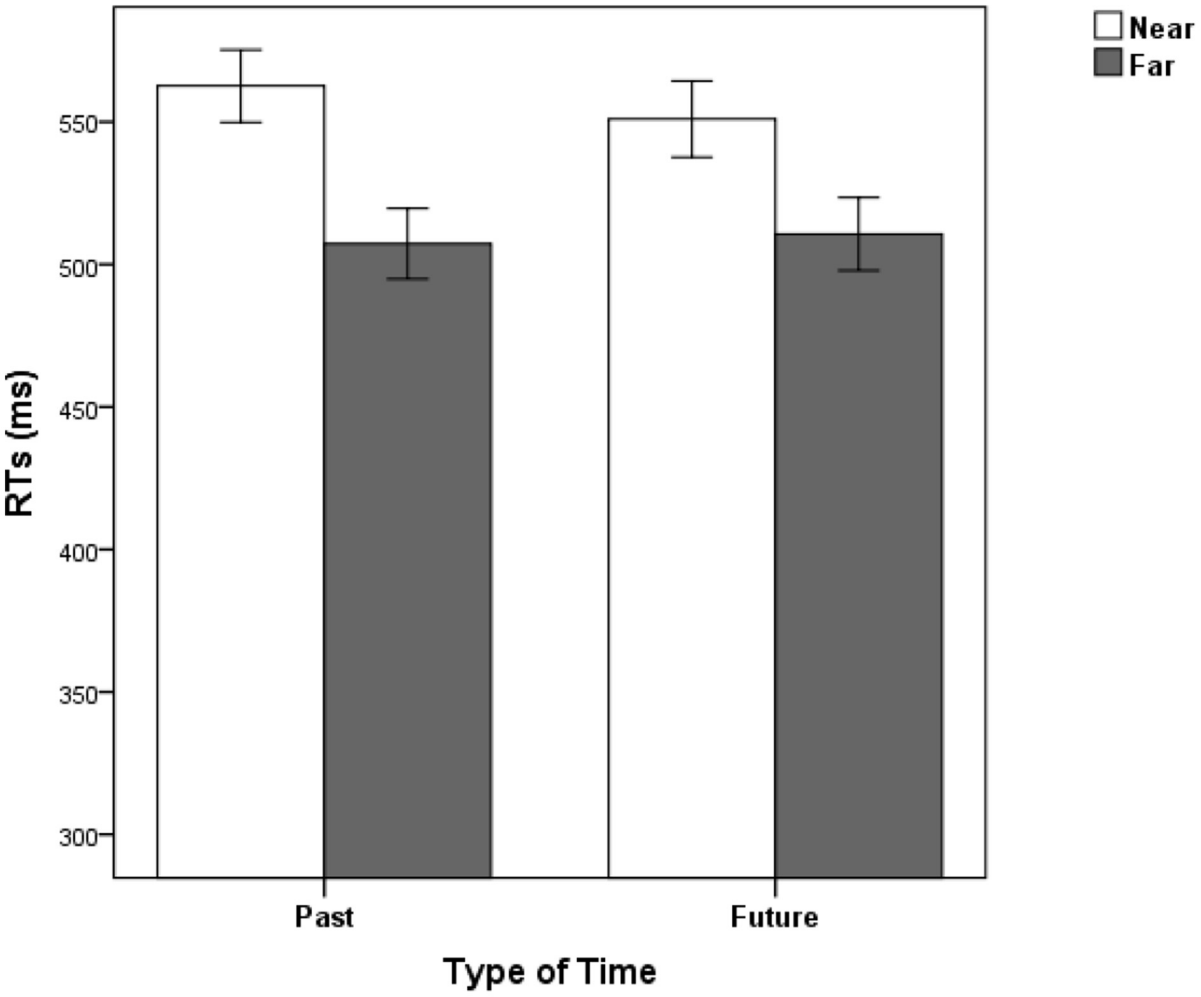
**Mean RTs in Experiment 3 as a function of type of time and distance**. Error bars represent standard errors of the means.

The mean error rate in judging the time words was 2.56%. No significant effect was observed in the MANOVA concerning accuracy. The results of RTs indicated that the main effect of response congruence was significant, *F*_(1, 34)_ = 13.09, *p* < 0.001, partial η^2^ = 0.29. The main effect of temporal distance was significant, *F*_(1, 34)_ = 7.17, *p* = 0.011, partial η^2^ = 0.17. The main effect of type of time words was not significant, *F*_(1, 34)_ = 1.64, *p* > 0.05. The interaction between temporal distance and type of time words was significant (See Figure 3), *F*_(1, 34)_ = 5.51, *p* = 0.025, partial η^2^ = 0.14. No other interactions were significant, *p*s > 0.05. Simple effect analysis revealed that the distance effect was greater for past time, *F*_(1, 34)_ = 9.69, *p* = 0.004; smaller for future time, *F*_(1, 34)_ = 4.74, *p* = 0.036.

The results of Experiment 3 revealed a typical STARC effect as in Experiments 1, 2. Past time words were responded faster with the left key, whereas future time words were responded faster with the right key. Moreover, a significant distance effect was observed. The time words of near distance (yesterday and tomorrow) were responded slower than far distance (last year and next year). Most importantly, there was an interaction between temporal distance and type of time words. The distance effect was greater for the past than for the future. These results further suggest that the spatial representations of past and future are asymmetric in the mental time line and that spatial representation of the past seems to be stronger than that of future.

However, some characteristics of time words, such as familiarity, could be confounded with temporal distance in Experiment 3. Separately we found that time words of near distance (yesterday or tomorrow) were more familiar than time words of far distance (last year or next year) in Experiment 3 through a questionnaire. Since familiar words were usually responded faster than unfamiliar words, it seemed that this distance effect could not be explained by familiarity of words. Nonetheless, we ran another experiment to balance the familiarity of time words in different distance.

## Experiment 4

Experiment 4 was designed to balance the familiarity of time words in different distance. Both familiar and unfamiliar time words were chosen in near and far distance condition. Moreover, we changed the response way from left-right direction to an orthogonal up-down direction in the keyboard. If distance effects of past and future were actually different, the way of response would not affect it.

### Methods

#### Participants

Eighteen undergraduate students (6 male and 12 female) from Central China Normal University participated in the experiment for course credits. All participants signed a consent form according to the requirements of Institutional Review Board of CCNU. They were 19.2 years old on average (range 18 to 20). All participants were naive to the purpose of the experiment.

#### Stimuli and apparatus

Thirty two Chinese time words were used. Sixteen words were near distance time words, 8 referring to past time of yesterday (e.g., yesterday morning, yesterday evening, etc.) and the other 8 referring to future time of tomorrow (e.g., tomorrow morning, tomorrow evening, etc.). Sixteen words were far distance time words, 8 referring to past time of last year (e.g., Labor Day of last year, National Day of last year, etc.) and the other 8 referring to future time of next year (e.g., Labor Day of next year, National Day of next year, etc.). In a pre-experimental questionnaire investigation, 46 subjects rated the familiarity of 32 time words from 1 (unfamiliar) to 5 (familiar). The results showed that the familiarity of time words in different temporal distances were not significantly different (*M*_near_ = 3.48 vs. *M*_far_ = 3.31), *F*_(1, 45)_ = 1.81, *p* = 0.19.

#### Experimental design

We used a 2 × 2 × 2 within-subjects design. Three independent variables were time distance (near vs. far), type of time words (past vs. future), response key (up arrow vs. down arrow). RTs and accuracy rates were dependent variables.

#### Procedure

The procedure was similar to the previous experiments. Participants were required to judge whether the time of word was earlier or later than present. For example, Yesterday morning or National Day of last year was earlier than present. In one session, the participant pressed up key (up arrow in the keyboard) if earlier and pressed the down key (down arrow in the keyboard) if later. In the other session participants were required to respond in the opposite way. The order of the two sessions was counterbalanced across participants. The participants were required to respond as fast and accurately as possible using the middle finger of the right hand only. Each session included 10 trials of practice and 8 blocks of 320 trials in the formal experiment.

#### Data analysis

Data analysis was the same as in Experiment 1.

### Results and discussion

**Figure 4:**
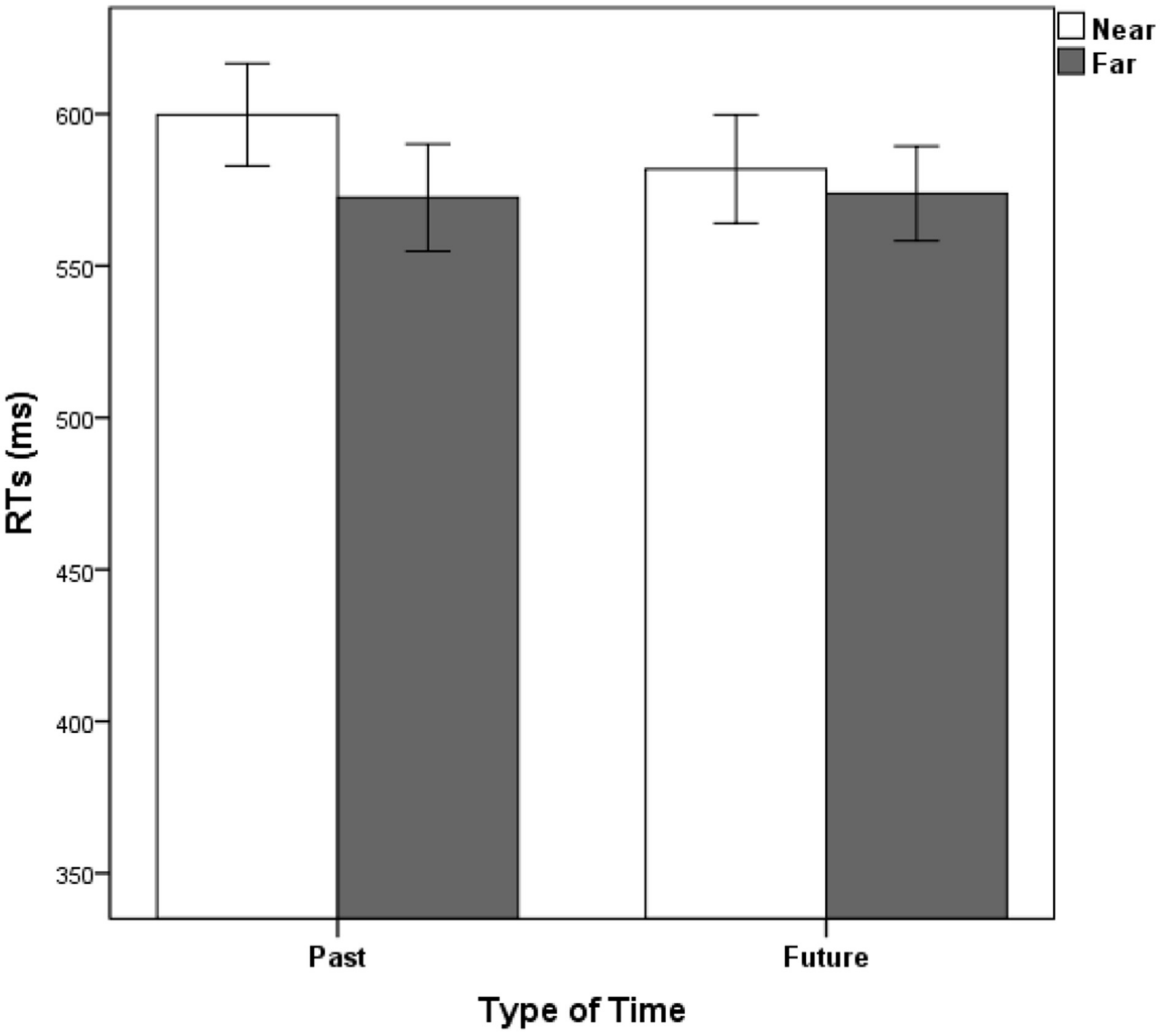
**Mean RTs in Experiment 4 as a function of type of time and distance**. Error bars represent standard errors of the means.

The mean error rate in judging the time words was 3.13%. No significant effect was observed in the MANOVA concerning accuracy. The results of RTs indicated that the main effect of time distance was significant, *F*_(1, 17)_ = 29.04, *p* < 0.001, partial η^2^ = 0.63. The interaction between time distance and type of time words was significant (See Figure 4), *F*_(1, 17)_ = 8.05, *p* = 0.011, partial η^2^ = 0.32. All other main effects and interactions were not significant, *p*s > 0.05. As the interaction between time distance and type of time words was significant, a simple effect analysis revealed that distance effect for past time, *F*_(1, 17)_ = 24.57, *p* < 0.001; and a smaller effect for future time, *F*_(1, 17)_ = 4.58, *p* = 0.047.

The results of Experiment 4 were similar as in Experiment 3. After controlling the familiarity of time words, the distance effect was still observed. The time words of near distance (yesterday and tomorrow) were responded slower than words of far distance (last year and next year). More important, the distance effect of past was also greater than that of future. Again, this result suggests that spatial representations of past and future were asymmetric in the mental time line and the spatial representation of the past seemed stronger than that of the future.

## General discussion

Previous findings supported that representation of time flows from past to future in a continuous spatial line with a left-to-right orientation. The present study provided the first empirical evidence for a fundamental characteristic of the mental time line: Are the spatial representations of past and future symmetric in the mental time line?

In Experiment 1, we compared STARC effects under near past and near future conditions. As expected, a typical STARC effect was observed. Early time was responded faster with the left key, whereas late time was responded faster with the right key. Moreover, STARC effects were the same between yesterday and tomorrow. This result indicated that spatial representations of past and future were symmetric in near past and near future in the mental time line. In Experiment 2, STARC effects were further compared under distant past and distant future condition. However, the STARC effect was only observed in the distant past condition, not in the distant future condition. This result showed that spatial representations of distant future and distant past were asymmetric in the mental time line and the spatial representation of past seemed stronger than that of future, as the STARC effect disappeared in distant future. Therefore, it seemed that past and future in the mental time line were symmetric in near space, but not in distant space.

In Experiments 3, 4, distance effects were compared under past and future conditions. Results showed that there were both a significant STARC effect and a distance effect in Experiment 3. Past time words were responded faster with the left key, whereas future time words were responded faster with the right key. When compared with the present, time points in the far distance (last year or next year) were responded faster than in the near distance (yesterday and tomorrow). Moreover, the distance effect in the past was greater than in the future. The same result was observed even when the response was changed to an orthogonal direction in Experiment 4. These results about distance effects support the idea that past and future are represented asymmetrically in the mental time line. Again, the spatial representation of the past seemed stronger than that of the past, as the distance effect for the future was smaller than that of the past.

These findings revealed that the mental time line is not evenly distributed and the past and future were asymmetric in the mental time line. According to our results, the STARC effect was significant for the distant past, but not for the distant future. And the distance effect was stronger for the past than for the future. Why is past different from future? A possible reason is that the past is more concrete or clear than the future. The past is time we have actually experienced. It is true and available for us. We can store the past information in our memory and retrieve it. The construction of the representation of past could be based on real events. However, the future is not yet true. It is obscure, abstract and fictional for us. The construction of the representation of future could only be based on fictional events.

This temporal asymmetry was in line with findings in some other paradigms. For example, Vallesi et al. (2008) found a stronger leftward representation of short durations than rightward representation of long durations in their fourth experiment at least numerically, suggesting a similar asymmetrical effect though no further statistics were provided in this literature. In a neuroimaging study, Okuda et al. (2003) found that anteromedial frontal pole and medial temporal areas showed a significant effect of temporal distance from the present. Specifically, the increase in brain activity in the left parahippocampal gyrus (BA 36) from the near future task to the far future task was smaller than that from the near past task to the far past task. It suggested that the distance effect was smaller for the future than for the past task. Addis and Schacter (2008) found that representations of past events were associated with more specific details than representations of future events (See also D'Argembeau and van der Linden, 2004, 2006; Wang et al., 2011). Future events were also more prototypical than past events (Kane et al., 2012). These findings supported that the representation of past was more concrete or clear than that of future.

Although the future is different from the past, it is similar in that the past and future are represented as a spatial line. STARC effects and distance effects were found for both past and future. These findings were consistent with constructive episodic simulation hypothesis (Schacter and Addis, 2007a,b). Since future is what we have never experienced before, how do we construct representations that we never truly experienced? Schacter and Addis (2007a,b) thought that one important function of constructive episodic memory is to allow individuals to simulate or imagine future episodes. We construct the future based on the past that we have experienced. Therefore, there should be considerable overlap in the psychological and neural processes involved in remembering the past and imagining the future. Neuroimaging evidence from Mental Time Traveling (MTT) supported that the underlying neural mechanisms for past and future were similar. Remembering the past and imagining the future may activate the same brain areas (Okuda et al., 2003; Schacter et al., 2007; Szpunar et al., 2007). These findings have led to the concept of the prospective brain and an idea that a crucial function of the brain is to use stored information to imagine, simulate and predict possible future events (Schacter et al., 2007).

The present study further indicated that the internal spatial representation of past or future seemed to be unevenly-distributed in the mental time line. Taking the results of Experiments 1, 2 together, the STARC effect was significant in tomorrow (near future) condition but not in next year (distant future) condition. Thus, the spatial representation of the near future seemed stronger than that of distant future in the mental time line. This finding was consistent with some studies about a loglinear characteristic of mental time. When participants were asked to judge whether an event of past or future was before or after an imagined “location” on the time line, the reaction time of this “self-projection” decreased logarithmically as the temporal distance between this imagined location and the location of another imagined event from the time line increased (Arzy et al., 2009). In addition, logarithmic curves were also found to fit the relation between temporal distance and memory, as the distribution of the correct recall of events from different points in time was logarithmic (Rubin and Schulkind, 1997; Spreng and Levine, 2006).

This logarithmic pattern suggested that time points (past or future) near the present were relatively sparse, and time points far from the present were relatively dense in the mental time line. In other words, if a set of two time points is near the present, the spatial distance would be larger, as the reaction times decrease sharply with the increase of the temporal distance; if the set of two time points was far from the present, the spatial distance would be smaller, as the reaction times decrease slowly with the increase of the temporal distance. Interestingly, a logarithmic pattern was also found in the mental number line. Humans map numbers into space line in logarithmic scaling (Dehaene and Cohen, 1995; Siegler and Booth, 2004; Dehaene et al., 2008). The mechanisms of processing time and number are similar, in line with the A Theory of Magnitude, i.e., ATOM (Walsh, 2003; Bueti and Walsh, 2009). Nonetheless, we should be cautious with these inferences and further research is needed on this logarithmic pattern of spatial representation in the mental time line.

Finally, it was worth noting that culture may play an important role on the representations of past and future. For instance, reading and writing habits can change the direction of the mental time line. The direction is from left to right in English or Italian speakers, whereas the direction is from right to left for Arabic or Hebraic speakers (Fuhrman and Boroditsky, 2010; Ouellet et al., 2010b; Vallesi et al., 2014). Most importantly, culture may shape the characteristics of the mental time line. Westerners exhibit greater episodic specificity than East Asians (Wang et al., 2011). The spatial representations of mental time for Westerners might be stronger than those of East Asians. Age and gender may also influence the representations of past and future. Older adults generated fewer internal details than younger adults for both past and future events (Addis and Schacter, 2008). Women exhibit greater episodic specificity than men for both past and future events (Wang et al., 2011).

In summary, the present study provided the first empirical evidence for the characteristics of the mental time line. Time points are not evenly distributed in the mental time line. The differences on STARC effect and distance effect supported that the spatial representations of past and future are asymmetric in the mental time line. And the spatial representation of past seemed stronger than that of future. Importantly, future studies should focus more on the characteristics of internal spatial representation of past or future (e.g., logarithmic pattern) and how the culture and some other factors shape the characteristics of the mental time line.

### Conflict of interest statement

The authors declare that the research was conducted in the absence of any commercial or financial relationships that could be construed as a potential conflict of interest.
