# Supplementary material for: Are past and future symmetric in mental time line?
Source: Front Psychol. 2015 Feb 26;6:208. doi: 10.3389/fpsyg.2015.00208 (PMC4341513; doi:10.3389/fpsyg.2015.00208)
Supplement: Supplementary file 1 [file DataSheet1.DOCX]

Appendix

Stimuli in Experiment1

Yesterday

昨天清晨 昨天黎明 昨天早晨 昨天上午 昨天下午 昨天傍晚 昨天夜里 昨天深夜

Tomorrow

明天清晨 明天黎明 明天早晨 明天上午 明天下午 明天傍晚 明天夜里 明天深夜

Stimuli in Experiment 2

Last year

去年元宵 去年清明 去年五一 去年端午 去年中秋 去年国庆 去年重阳 去年圣诞

Next year

明年元宵 明年清明 明年五一 明年端午 明年中秋 明年国庆 明年重阳 明年圣诞

Stimuli in Experiment 3

Near space (Yesterday and tomorrow)

Past 昨天清晨 昨天上午 昨天下午 昨天深夜

Future 明天清晨 明天上午 明天下午 明天深夜

Far space (Last year and Next year)

Past 去年元宵 去年五一 去年国庆 去年圣诞

Future 明年元宵 明年五一 明年国庆 明年圣诞

Stimuli in Experiment 4

Near space (Yesterday and tomorrow)

Past昨天清晨 昨天黎明 昨天早晨 昨天上午 昨天下午 昨天傍晚 昨天夜里 昨天深夜

Future 明天清晨 明天黎明 明天早晨 明天上午 明天下午 明天傍晚 明天夜里 明天深夜

Far space (Last year and Next year)

Past去年元宵 去年清明 去年五一 去年端午 去年中秋 去年国庆 去年重阳 去年圣诞

Future明年元宵 明年清明 明年五一 明年端午 明年中秋 明年国庆 明年重阳 明年圣诞
